# Supplementary material for: Complexome profiling of the Chlamydomonas psb28 mutant reveals TEF5 as an early PSII assembly factor
Source: Plant Cell. 2025 Mar 17;37(6):koaf055. doi: 10.1093/plcell/koaf055 (PMC12164590; doi:10.1093/plcell/koaf055)
Supplement: koaf055_Supplementary_Data [file koaf055_supplementary_data.zip › psb28_interactive.zip!/plots/intensity/qualityPlots/peptides/RIP1_Cre01.g051900.t1.2.html]

### Cre01.g051900.t1.2

Trivial name: RIP1  
SeqCoverage [median]: 37.40  
Mass: 28.51 kDa  
  
 

| Sequence | MissedCleavage | Mass [Da] | wtIntensity | muIntensity | Score |
| --- | --- | --- | --- | --- | --- |
| EGPAPYNLEVPEYR | 0 | 1632.78 | 30311.02 | 78361.46 | 221.09 |
| FLYASAAR | 0 | 897.47 | 55971.98 | 67087.13 | 120.49 |
| FPPGTEGRPFAYFVQTGGR | 1 | 2083.03 | 9493.43 | 28275.88 | 65.47 |
| FTEGQK | 0 | 708.34 | 27675.14 | 27303.42 | 138.61 |
| FTEGQKVVIG | 1 | 1076.59 | 0.00 | 1065.38 | 109.11 |
| HRTDAEIAQSAEVALSELRDPQK | 2 | 2563.30 | 5865.97 | 11167.62 | 88.09 |
| HRTDAEIAQSAEVALSELRDPQKDVDR | 3 | 3048.52 | 21367.55 | 39863.36 | 112.99 |
| IREGPAPYNLEVPEYR | 1 | 1901.96 | 2335.00 | 9415.41 | 97.90 |
| LSMAPTPYIK | 0 | 1119.60 | 50383.78 | 50040.51 | 193.10 |
| LSMAPTPYIKYDEHNHK | 1 | 2042.99 | 0.00 | 905.55 | 43.79 |
| PFAYFVQTGGR | 0 | 1241.62 | 2967.68 | 2158.14 | 112.84 |
| RFPPGTEGRPFAYFVQTGGR | 2 | 2239.13 | 1487.43 | 3168.24 | 45.09 |
| TDAEIAQSAEVALSELRDPQK | 1 | 2270.14 | 3943.74 | 0.00 | 77.39 |
| TDAEIAQSAEVALSELRDPQKDVDR | 2 | 2755.36 | 10970.06 | 0.00 | 122.39 |
|  |  |  | 222772.8 | 318812.1 |  |
